# EIN3-binding F-box protein SlEBF3 modulates resistance against *Botrytis cinerea* and carotenoid biosynthesis by degradation of BBX20 in tomato

**DOI:** 10.1093/hr/uhaf219

**Published:** 2025-08-22

**Authors:** Zhuo Gao, Heng Deng, Xiaoqing He, Yanpeng Yin, Chengpeng Yang, Tianhao Mao, Jinyan Guo, Mondher Bouzayen, Mingchun Liu, Mengbo Wu

**Affiliations:** Key Laboratory of Bio-Resource and Eco-Environment of Ministry of Education, College of Life Sciences, Sichuan University, Chengdu, Sichuan 610065, China; School of Life Science and Engineering, Southwest University of Science and Technology, Mianyang 621010, China; Key Laboratory of Bio-Resource and Eco-Environment of Ministry of Education, College of Life Sciences, Sichuan University, Chengdu, Sichuan 610065, China; Key Laboratory of Bio-Resource and Eco-Environment of Ministry of Education, College of Life Sciences, Sichuan University, Chengdu, Sichuan 610065, China; Key Laboratory of Bio-Resource and Eco-Environment of Ministry of Education, College of Life Sciences, Sichuan University, Chengdu, Sichuan 610065, China; Key Laboratory of Bio-Resource and Eco-Environment of Ministry of Education, College of Life Sciences, Sichuan University, Chengdu, Sichuan 610065, China; Key Laboratory of Bio-Resource and Eco-Environment of Ministry of Education, College of Life Sciences, Sichuan University, Chengdu, Sichuan 610065, China; Laboratoire de Recherche en Sciences Végétales-Génomique et Biotechnologie des Fruits-UMR5546, Université de Toulouse, CNRS, UPS, Toulouse-INP, Toulouse, France; Key Laboratory of Bio-Resource and Eco-Environment of Ministry of Education, College of Life Sciences, Sichuan University, Chengdu, Sichuan 610065, China; Key Laboratory of Bio-Resource and Eco-Environment of Ministry of Education, College of Life Sciences, Sichuan University, Chengdu, Sichuan 610065, China

## Abstract

EIN3 binding F-box (EBF) proteins have been reported to play important roles in ethylene signaling pathway by mediating the ubiquitin-dependent degradation of EIN3-Like (EIL) proteins, but little is known about their roles in postharvest disease resistance. Here, we showed that SlEBF3 confers resistance against *Botrytis cinerea* by ubiquitin-mediated degradation of SlBBX20. Overexpression of *SlEBF3* enhanced resistance to *B. cinerea* and increased the expression levels of genes related to PR (pathogenesis-related) and JA (jasmonic acid) in tomato, while knockdown of *SlEBF3* does not affect tomato resistance to *B. cinerea*. Further study demonstrated that SlEBF3 interacts with SlBBX20 the interaction between SlEBF3 and SlBBX20 promotes SlBBX20 degradation via the 26S proteasome, which confers enhanced resistance to *B. cinerea* through the JA signaling pathway mediated by the SlBBX20-SlMYC2-SlMED25 module. Meanwhile, SlEBF3 extends fruit shelf life by remodeling cell wall composition and promoting cuticular accumulation. Additionally, SlEBF3 is involved in carotenoid metabolism regulation by interacting with SlBBX20, SlRIN, SlFUL1, and SlTAGL1, which is independent of the degradation of EIL proteins. Overall, this study revealed the molecular mechanism by which *SlEBF3* responds to JA signaling to regulate *B. cinerea* resistance, enriched the roles of *SlEBF3* in the regulatory network of carotenoids metabolism, and provided new insights into the extension of fruit shelf life.

## Introduction

*Botrytis cinerea* is a necrotrophic phytopathogenic fungus that can infect hundreds of plants and cause significant economic and agricultural losses worldwide [1, 2]. As a leading commercial vegetable crop with global cultivation significance, tomato (*Solanum lycopersicum*) exhibits heightened susceptibility to *B. cinerea* infestation throughout its maturation phase. This pathogen causes gray mold disease, leading to significant postharvest losses and reduced yield [3, 4]. The use of chemical fungicides is currently an effective strategy for controlling *B. cinerea*, while overuse poses new threats to ecosystems and consumer health [5]. Consequently, understanding the molecular mechanisms of fruit resistance to *B. cinerea* is essential for the development of eco-friendly strategies for disease control, thereby preserving the postharvest quality and economic value of fruits.

The phytohormone jasmonic acid (JA) orchestrates diverse developmental pathways essential for plant morphogenesis and enhances defense responses against physical injury and microbial pathogenesis [6–9]. After decades of research, the molecular mechanism of JA signaling, consisting of multiple interacting proteins, has been elucidated. The bHLH transcription factor MYC2 serves as a master regulator, integrating diverse JA-responsive pathways [10]. Without JA, JAZ proteins interact with the corepressor TOPLESS to form transcriptional repression complexes. These complexes inhibit MYC2-dependent gene activation by interfering with the ability of transcription factors to bind target DNA. When the bioactive JA ligand jasmonoyl-isoleucine (JA-Ile) is present, JAZ proteins bind with CORONATINE-INSENSITIVE1 (COI1), the F-box component of the SCFCOI1 E3 ligase complex. This JA-Ile-dependent interaction initiates the polyubiquitination of JAZ proteins and their subsequent proteasomal degradation, thereby relieving the inhibition of MYC2. Subsequently, MYC2 interacts with the mediator subunit MED25 to form a functional transcriptional complex, which activates the expression of JA-regulated genes such as *SlJA2L* (JA2-Like) and ERF.C3 [10–15]. BBX proteins are characterized by their zinc-finger domains and represent an important family of transcriptional regulators involved in diverse physiological processes. Recent studies demonstrate that BBX20 both regulates anthocyanin biosynthesis in fruit while suppressing JA-mediated defense responses through inhibiting the accumulation of MED25, consequently reducing resistance to *B. cinerea*. [16].

As a crucial phytohormone, ethylene (ET) serves dual biological functions not only by orchestrating the maturation of climacteric fruits but also by exerting significant influence on plant defense mechanisms and immune-related processes [17–21, 23]. The *SlERF* members has been extensively researched on resistance to *B. cinerea*. The susceptibility of fruit to *B. cinerea* increased with the knockout of *SlERF.C1*, whereas its overexpression (OE) improved resistance [21]. Silencing of *SlERF.A1*, *SlERF.A3 (Pit4)*, *SlERF.B4*, and *SlERF.C3 (Pit5)* via virus-induced gene silencing (VIGA) increased susceptibility to *B. cinerea* in tomato leaves [24]. Ethylene Insensitive3 Like1(*EIL1*), an upstream of ethylene response factors (*ERFs*), targets and activates the JA metabolizing gene *SlCYP94C1,* thereby negating JA-mediated resistance [25]. While *SlERFs* and *SlEIL1* are well characterized in the defense against *B. cinerea*, the involvement of other ET signaling pathway members in the disease resistance is still uncertain.

Carotenoids are vital phytonutrients in tomato and their synthesis is mediated by various enzymes such as phytoene synthase (PSY) and phytoene desaturase among others. Tomato mutants with loss-of-function of *SlPSY1* produce yellow fruits indicating that *SlPSY1* plays a crucial role in regulating carotenoid synthesis [26, 27]. Transcription factors, including as SlBBX20, SlMYB72, SlMYB1, and HY5 have been shown to directly interact with the promoter of *SlPSY1* to regulate carotenoid synthesis [28–31]. During tomato ripening, key transcription factors such as RIN, FUL1, TAGL1, and NOR play important functions in the regulation of carotenoid metabolism and have been widely studied [32–35]. Our recent finding indicate that *SlEBF3* (EIN3 BINDING F-BOX 3) plays a role in climacteric fruit ripening by facilitating the degradation of EIL proteins [36]. However, the relationships between these EBF proteins and these key transcription factors regulating carotenoids synthesis are unknown.

This study reveals novel mechanisms of SlEBF3 in regulating tomato fruit resistance to *B. cinerea*, phytoene synthesis, and fruit ripening through interactions with BBX20, RIN, FUL1, and TAGL1. Additionally, SlEBF3 shows functional redundancy with other SlEBF genes in mediating resistance to *B. cinerea*, but exhibiting distinct regulatory effects on phytoene synthesis. Knockdown of SlEBF3 did not notably affect resistance to *B. cinerea*, but it significantly increased phytoene content compared to wild type (WT). OE of SlEBF3 markedly increased resistance to *B. cinerea*, accompanied by a notable reduction in phytoene content. In our study, we found that SlEBF3 responds to JA signaling through the degradation of SlBBX20 and thus regulates tomato resistance to *B. cinerea*. In addition, SlEBF3 interacts with RIN, FUL1, and TAGL1 to regulate phytoene synthesis and fruit ripening, while none of the above results were accomplished by degrading EIL. In conclusion, our study identified a novel function of SlEBF3 in regulating resistance against *B. cinerea* in tomato fruits and enriched the regulatory network of carotenoid synthesis and ripening in fruits, which provides a new strategy for maintaining postharvest fruit quality.

## Results

### *SlEBF3* is induced by *B. cinerea* and enhances resistance to *B. cinerea* infection in tomato fruits

**Figure 1:**
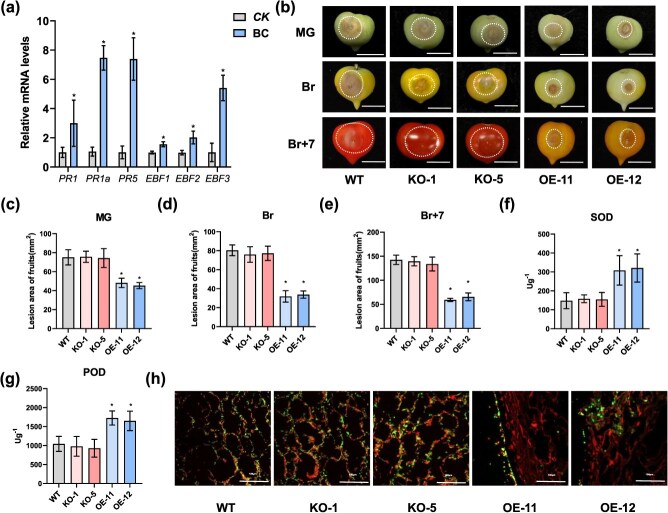
*SlEBF3* is induced by *B. cinerea* and enhances resistance against *B. cinerea* infection in tomato. (a) Relative transcript levels of pathogenesis-related (PR) genes and *SlEBFs* in response to *B. cinerea* infection. CK, blank potato dextrose agar (PDA) media. BC, *B. cinerea*. Relative mRNA levels of each gene in WT before *B. cinerea* infection were normalized to 1. Data are shown as means ± SD (*n* = 3). Asterisks indicate statistical significance using Student’s *t*-test, *P* < 0.05. *B. cinerea* infection were normalized to 1, and *SlActin* was used as an internal control. (b) Disease phenotype in WT, *SlEBF3*-KO lines, and *SlEBF3*-OE fruits after incubated with *B. cinerea*. Fruits were collected at MG, Br, and Br + 7 stages and then infected with *B. cinerea*. Photographs were taken at 48 h hpi/2 DPI. MG (mature green) stage, Br (Br stage), Br + 7, 7 d after the Br stage. Bar = 10 mm. (c–e) Lesion diameter in tomato fruits after infected with *B. cinerea* for 2 d at MG (c), Br (d), and Br + 7 (e) stages. Data are shown as means ± SD (*n* = 10). Asterisks indicate statistical significance using Student’s *t*-test, *P* < 0.05. (f, g) Antioxidant enzymatic activities of SOD (f) and POD (g) in fruits at 2 DPI with *B. cinerea*. Data are shown as means ± SD (*n* = 10). Asterisks indicate statistical significance using Student’s *t*-test, *P* < 0.05. (h) Fungal hyphae in planta were visible in the WGA-PI-stained pericarp from WT, *SlEBF3*-KO, and *SlEBF3*-OE fruits after infected with *B. cinerea* under the fluorescence microscopy at Br stages. Fungal hyphae show green fluorescence, cell walls of plant show magenta fluorescence (replacing red fluorescence with magenta). Bar = 100 μm.

Previous research has shown that SlEBF3 participates in regulating fruit maturation and carotenoid biosynthesis by interacting with EILs and repressing it accumulation. However, its involvement in the response to *B. cinerea* remains unclear. To investigate whether *SlEBFs* responds to *B. cinerea* infestation, RT-qPCR assay was performed after infesting WT plants in tomato leaves with *B. cinerea*. Similar with PR genes, *SlEBFs* were significantly upregulated (Fig. 1a), demonstrating that *SlEBFs* are typical *B. cinerea* response genes. Given that *SlEBF3* exhibited the most pronounced changes among the three EBF genes, we speculate that EBF3 plays a pivotal role in responding to *B. cinerea* infection (Fig. 1a). To analyze the function of SlEBF3 in *B. cinerea* resistance, we generated *SlEBF3* knockout (*SlEBF3*-KO) lines by CRISPR/Cas9 mediated genome editing technology and obtained two KO lines with 5-bp deletion and 16-bp insertion and resulting in truncated versions with 22 and 61 amino acids remaining, respectively (Fig. S1a). Two OE lines were used for further study (Fig. S1b). There was no effect on vegetable growth such as plant size (Fig. S2a and b). Apart from delayed fruit ripening in *SlEBF3*-OE lines (Fig. S2c and d), no significant differences in fruit morphology or pericarp thickness were observed among the WT, *SlEBF3*-KO, and *SlEBF3*-OE lines (Fig. S2e–g).

To assess the potential role of *SlEBF3* in conferring resistance to *B. cinerea* during fruit development, we collected WT, *SlEBF3*-KO, and *SlEBF3*-OE fruits at MG (mature green), Br (breaker), and Br + 7 stages and inoculated them with *B. cinerea*. After 48 h post-inoculation (hpi), the area of the lesions was measured (Fig. 1b). As shown in Fig. 1, the mean lesion size in SlEBF3-OE fruits was significantly smaller than that in WT fruits at the MG, Br, and Br + 7 stages, with no differences observed between WT and *SlEBF3*-KO across these stages (Fig. 1c–e). We quantified the activities of two important antioxidant enzymes, superoxide dismutase (SOD), and peroxidase (POD). *SlEBF3*-OEs fruits demonstrated notably higher SOD and POD activities compared to WT fruits, whereas SlEBF3-KO fruits did not display any significant differences in these activities relative to WT fruits (Fig. 1f and g). To further assess the severity of the disease, we analyzed the spread of *B. cinerea* hyphae in infected fruits by WGA-PI staining. Intense green fluorescence was distributed along the cell wall and/or in the intercellular spaces in the tissues of WT and *SlEBF3*-KO fruits, whereas it was significantly reduced in the tissues of *SlEBF3*-OE fruits (Fig. 1h). These findings indicated that compared with WT and *SlEBF3*-KO, *SlEBF3*-OE fruits are less susceptible to *B. cinerea* infection. These findings indicate that *SlEBF3* enhances resistance to *B. cinerea* infection.

### *SlEBF3* enhances resistance to *B. cinerea* in tomato leaves

**Figure 2:**
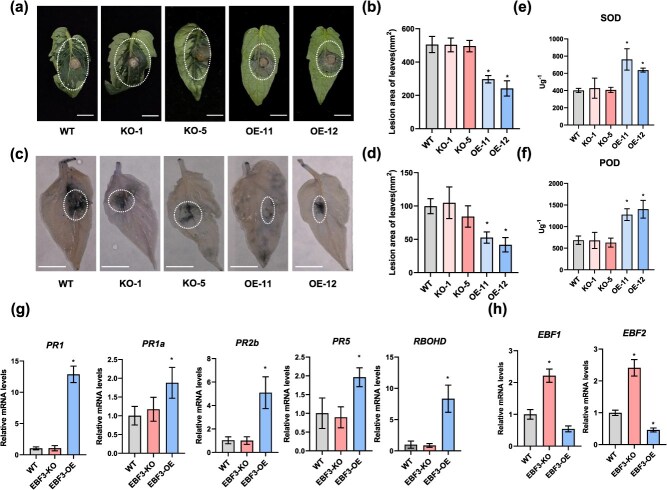
*SlEBF3* enhances resistance to *B. cinerea* in tomato leaves. (a) Disease symptom on detached leaves 4 days after infected with *B. cinerea* on WT, *SlEBF3*-KO, and *SlEBF3-*OE lines. Bar = 10 mm. (b) Lesion diameter in leaves at 4 DPI. At least 10 leaves from each line were infected with *B. cinerea* for lesion size measurement. Data are shown as means ± SD (*n* = 10). Asterisks indicate statistical significance using Student’s *t*-test, *P* < 0.05. (c) Representative images of trypan blue staining for cell death in indicated tomato leaves at 2 DPI with *B. cinerea*. Bar = 10 mm. (d) Lesion diameter in leaves at 2 DPI. At least 5 leaves from each line were infected with *B. cinerea* for lesion size measurement. Data are shown as means ± SD (*n* = 5). Asterisks indicate statistical significance using Student’s *t*-test, *P* < 0.05. (e, f) Antioxidant enzymatic activities of SOD (e) and POD (f) in leaves at 2 DPI with *B. cinerea*. Data are shown as means ± SD (*n* = 3). Asterisks indicate statistical significance using Student’s *t*-test, *P* < 0.05. (g) Relative expression levels of disease resistance-related genes *SlPR1*, *SlPR1a*, *SlPR2b*, *SlPR5*, and *SlRBOHD* in WT, *SlEBF3*-KO, and *SlEBF3*-OE lines. (h) Relative expression levels of SlEBF1, SlEBF2 in WT, SlEBF3-KO, and SlEBF3-OE lines after infected with *B. cinerea*. The transcript levels in WT were normalized to 1, and *SlActin* were used as an internal standard. Data are shown as means ± SD (*n* = 3). Asterisks indicate statistical significance using Student’s *t*-test, *P* < 0.05.

We evaluated the transgenic plant’s resistance to *B. cinerea* in leaves. Four days after infestation, the lesion area on these leaves was measured. The mean lesion area of *SlEBF3*-OE leaves was significantly smaller compared to WT leaves, whereas no significant difference was observed between *SlEBF3*-KO and WT leaves (Fig. 2a and b). To visualize the difference in resistance between *SlEBF3* transgenic plants and WT, detached leaves inoculated with *B. cinerea* for two days were stained with trypan blue. *SlEBF3*-OE leaves had smaller areas of dead cells compared to WT, while *SlEBF3*-KO leaves showed no significant difference from WT leaves (Fig. 2c and d). After 48 hpi with *B. cinerea*, *SlEBF3*-OEs leaves exhibited increased SOD and POD activities compared to WT (Fig. 2e and f).

Meanwhile, the expression levels of disease resistance-related genes *SlPR1, SlPR1a, SlPR2b, SlPR5*, and *SlRBOHD* were significantly increased in *SlEBF3*-OEs lines (Fig. 2g). These findings suggest that *SlEBF3* positively influences tomato leaf resistance to *B. cinerea*, with its OE significantly enhances this resistance*.* The KO *SlEBF3* did not impact tomato resistance to *B. cinerea*, suggesting potential functional redundancy among the three *SlEBF3* genes in tomato. To verify this hypothesis, we examined the expression of *SlEBF3* homologous family members *SlEBF1* and *SlEBF2* in *SlEBF3*-KO and *SlEBF3*-OE fruits after inoculation with *B. cinerea*. The results showed that *SlEBF1* and *SlEBF2* were significantly upregulated in *SlEBF3*-KO and downregulated in *SlEBF3*-OE after inoculation with *B. cinerea* (Fig. 2h), which demonstrated the functional redundancy of *SlEBFs* in regulating *B. cinerea* resistance.

### *SlEBF3* affects the expression of disease resistance-related genes in tomato fruit

**Figure 3:**
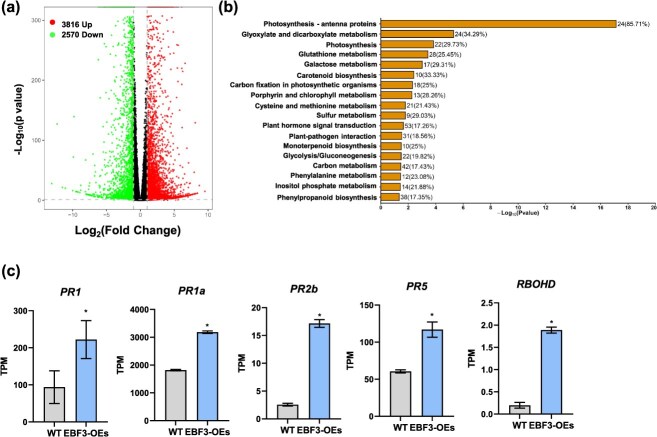
RNA-Seq analysis of WT and *SlEBF3*-OE fruits. (a) Volcano diagrams of DEGs in WT and *SlEBF3*-OE fruits at 2 DPI at Br stage. The block colored in red and green means the up-regulated genes and downregulated genes, respectively. (b) KEGG analysis of 6386 DEGs in WT and *SlEBF3*-OE fruits. (c) Expression levels of disease resistance–related genes *SlPR1*, *SlPR1a*, *SlPR2b*, *SlPR5*, and *SlRBOHD* in WT and *SlEBF3*-OE fruits. Data are shown as means ± SD (*n* = 3). Asterisks indicate statistical significance using Student’s *t*-test, *P* < 0.05.

To better understand the potential molecular mechanism by which *SlEBF3* contributes to *B. cinerea* resistance, we performed RNA-Seq on WT and *SlEBF3*-OE fruits that were harvested at the Br stage and infected with *B. cinerea* for 48 h, with three replicates per sample. In the comparison between *SlEBF3*-OE and WT fruits, a total of 6386 differentially expressed genes (DEGs) were identified, with 2570 genes were downregulated and 3816 genes upregulated, were found in *SlEBF3*-OE vs WT fruits (Fig. 3a; Dataset S2). KEGG analysis revealed that SlEBF3-OE influences pathways associated with glyoxylate and dicarboxylate metabolism, glutathione metabolism, plant hormone signal transduction and plant-pathogen interaction were affected by *SlEBF3*-OE in *B. cinerea*-infected fruits (Fig. 3b; Dataset S3). In alignment with the disease progression, the expression levels of PR genes such as *SlPR1*, *SlPR1a*, *SlPR2b*, and *SlPR5* and defense related (DR) genes such as Respiratory burst oxidase homolog D (*SIRBOHD*) were increased significantly in *SlEBF3*-OE fruits compared with WT following *B. cinerea* infection (Fig. 3c).

### *SlEBF3* is induced by JA and positively regulates JA signaling

**Figure 4:**
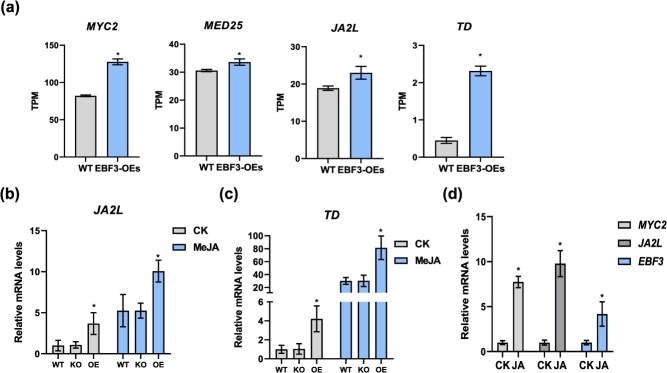
*SlEBF3* is induced by JA and positively regulates JA signalling. (a) Relative transcript levels of *SlMYC2*, *SlMED25*, *SlJA2L*, and *SlTD* genes in WT and *SlEBF3*-OE fruits at 2 DPI at Br stage. (b, c) Relative expression of *SlJA2L* (b) and SlTD (c) in WT, *SlEBF3*-KO, and *SlEBF3*-OE lines after MeJA treatment. Expression levels were normalized to untreated WT controls (set as 1). Data represent mean ± SD (*n* = 3). Asterisks indicate statistical significance using Student’s *t*-test, *P* < 0.05. (d) Relative expression of *SlMYC2, SlJA2L*, and *SlEBF3* in WT after MeJA treatment. Expression levels of each gene in WT were normalized to 1. Data represent mean ± SD (*n* = 3). Asterisks indicate statistical significance using Student’s *t*-test, *P* < 0.05.

ET and JA can induce the expression of PR genes [37]. SlEBF3 plays a negative regulatory role in the ET signaling pathway; therefore, we hypothesize that the upregulation of PR genes in *SlEBF3*-OE fruits may be attributed to activation of the JA signaling pathway. Consequently, we investigated the expression profiles of JA signaling and response-related genes in *SlEBF3*-OE fruits at 2 d post-inoculation (DPI). Transcriptomic analysis revealed substantial transcriptional upregulation of *SlMYC2*, *SlMED25*, *SlJA2L*, and *SlTD* (Fig. 4a). We used RT-qPCR to examine the transcript levels of relevant genes in fruits from WT, SlEBF3-KO, and SlEBF3-OE lines to determine whether SlEBF3 influences tomato disease resistance through JA signaling. The findings indicated that *SlEBF3*-OE lines exhibited increased transcription of *SlJA2L* and *SlTD* compared to WT lines, whereas no transcriptional differences were detected between WT and *SlEBF3*-KO lines (Fig. 4b and c). Notably, the transcript levels of these genes were consistently higher in *SlEBF3*-OE lines than in WT and *SlEBF3*-KO lines (Fig. 4b and c). These findings indicate that *SlEBF3* promotes the expression of *SlJA2L* and *SlTD.* The expression levels of *SlJA2L* and *SlTD* increased in WT, *SlEBF3*-KO, and *SlEBF3*-OE lines after MeJA treatment (Fig. 4b and c).

To examine whether *SlEBF3* expression is responsive to JA, we tested the transcript level of *SlEBF3* in WT after treatment with MeJA. These findings indicated a significant up-regulation after JA treatment, which was consistent with the trend of changes in the JA-responsive genes *SlMYC2* and *SlJA2L* indicating that *SlEBF3* can be induced by JA (Fig. 4d). These results suggest that *SlEBF3* is induced by JA and positively regulates JA signalling.

### SlEBF3 interacts with SlBBX20 and attenuates its suppression of SlMED25-SlMYC2 activation of *SlJA2L*

**Figure 5:**
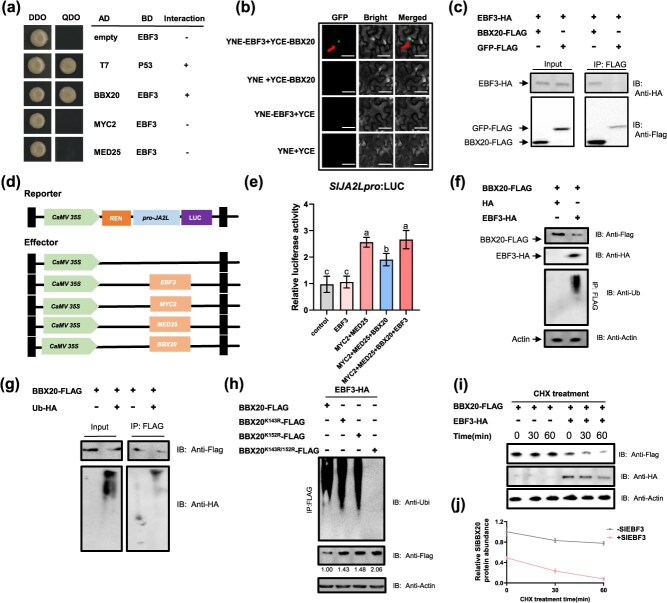
SlEBF3 interacts with SlBBX20 and attenuate its suppression of SlMED25-SlMYC2 activation of SlJA2L. (a) Y2H assay of interaction between SlEBF3 and SlBBX20, SlMYC2, and SlMED25. SlBBX20, SlMYC2, and SlMED25 were used as bait, SlEBF3 was used as prey. Interactions between P53 and T7 and between SlEBF3-BD and empty AD were used as positive and negative controls, respectively. (b) BiFC assay of interaction between SlEBF3 and SlBBX20. Bar = 50 μm. (c) *In vivo* Co-IP assays of SlEBF3 with SlBBX20. SlBBX20-FLAG was co-infiltrated with SlEBF3-HA in *N. benthamiana* leaves by Agrobacterium-mediated infiltration. Total proteins were extracted and immunoprecipitated with anti-FLAG antibody. GFP-FLAG was used as negative control. Immunoblots were probed with anti-FLAG antibody to detect SlBBX20 and anti-HA antibody to detect SlEBF3. (d) A schematic illustration of the LUC reporter and effector constructs used in transient expression assays. (e) Influence of SlEBF3-62sk on the expression of SlJA2L in transient expression assays. The LUC/REN ratio represents the relative activity of the SlJA2L promoter. Data are shown as means ± SD (*n* = 3). Values marked with different letters indicate statistically significant differences as analyzed by a one-way ANOVA test from three independent biological replicate experiments, *P* < 0.05. (f) Effect of SlEBF3 on the protein stability of SlBBX20 and *in vivo* ubiquitination of SlBBX20 by SlEBF3. The SlBBX20-Flag was expressed in the presence (+) or absence (−) of SlEBF3-HA in *N. benthamiana* leaves. After 3 days, protein was extracted from tobacco leaves. The SlEBF3-HA and SlBBX20-FLAG proteins were detected by immunoblotting. Proteins were analyzed by immunoblotting with anti-FLAG, anti-HA, and anti-actin antibodies. Total proteins extracted from transformed leaves were immunoprecipitated with anti-flag beads, the ubiquitination was detected by anti-ubiquitin antibody. The arrow indicates a band containing the SlBBX20 protein. The numbers under the bands indicate the ratio of SlBBX20 protein to actin. HA is pBTEX-HA empty vector used as a negative control. (g) *In vivo* detection of ubiquitination of SlBBX20-FLAG by Co-IP. (h) Identification for SlBBX20 site ubiquitinated by SlEBF3 via *in vivo* ubiquitination assay. The two predicted lysine (K) sites were mutated individually and together to arginine (R). The SlBBX20-Flag, SlBBX20^K152R^-Flag, SlBBX20^K143R^-Flag, and SlBBX20^K143R^/^K152R^-Flag were expressed in the presence of SlEBF3-HA in *N. benthamiana* leaves and subjected to ubiquitination analysis as described in (d). (i, j) Degradation rate analysis of SlBBX20 in the presence (+) or absence (−) of SlEBF3. Co-expression of SlBBX20-Flag with or without SlEBF3-HA was performed in *N. benthamiana* leaves, followed by treatment with translation inhibitor CHX. The total proteins were extracted for immunoblotting with anti-FLAG and anti-HA antibody at an indicated time point after treatment. The intensity of protein bands was quantified using ImageJ. Data are shown as means ± SD (*n* = 3).

To elucidate the molecular mechanisms by which SlEBF3 modulates JA signaling, we conducted a yeast two-hybrid (Y2H) screening assay to identify potential interacting proteins. The results indicated that SlEBF3 interacts with SlBBX20, a negative regulator of JA signalling, but not with SlMYC2 or SlMED25 (Fig. 5a). Additionally, a bimolecular fluorescence complementation (BiFC) assay was conducted to validate the interaction between SlEBF3 and SlBBX20. Tobacco leaves were coinjected with *Agrobacterium tumefaciens* strains carrying plasmids for SlEBF3-YNE and SlBBX20-YCE expression. Three days post-injection, fluorescence was observed in the experimental group but not in the control group (Fig. 5b). We conducted a co-immunoprecipitation (Co-IP) assay to confirm the interaction between the two proteins. The results revealed that SlBBX20-FLAG co-immunoprecipitated with SlEBF3-HA, but not GFP-FLAG (Fig. 5c). Taken together, our results demonstrate that SlEBF3 interacts with SlBBX20 *in vitro* and *in planta*.

It has reported that SlMYC2-SlMED25 can promote resistance to *B. cinerea* by activating the transcription of *Sl*JA2L, and SlBBX20 negatively regulates JA signaling through inhibition of SlMED25 protein accumulation. A dual luciferase assay was performed to evaluate the impact of SlEBF3 on the transcriptional activation of the *SlJA2L* promoter by the SlBBX20-SlMED25-SlMYC2 complex. Full-length coding sequences of *SlEBF3*, *SlMYC2*, *SlMED25*, and *SlBBX20* were amplified and inserted into the pGreen-62SK plasmid as the effector and the promoter of the JA-responsive gene *SlJA2L* was fused to a Mini35S-LUC as a reporter (Fig. 5d). Tobacco leaves were co-infiltrated with *A. tumefaciens* strains containing the effector and reporter gene constructs and the LUC/REN ratio was measured after three days. The results showed that SlMED25-SlMYC2 could activate the *SlJA2L* promoter and SlBBX20 could repress this transcriptional activation activity, which is consistent with the findings of previous studies [10, 16] (Fig. 5e). We found that SlEBF3 suppress the inhibitory effect of SlBBX20 on SlMED25-SlMYC2 activation of *SlJA2L* and did not affect the activity of the *SlJA2L* promoter alone (Fig. 5e). Collectively, these results demonstrate that SlEBF3 physically interacts with SlBBX20 to repress its regulatory function.

### SlEBF3 promotes SlBBX20 degradation through ubiquitin-mediated proteasomal degradation

To investigate whether the interaction between SlEBF3 and SlBBX20 affects SlBBX20 transcription, we analyzed the expression level of *SlBBX20* in WT, *SlEBF3*-KO, and *SlEBF3*-OE lines. The results showed that SlEBF3 did not significantly affect SlBBX20 transcript levels (Fig. S3a). We subsequently examined the protein accumulation when *SlBBX20* was expressed alone or co-expressed with *SlEBF3*. And found that SlBBX20 protein levels were significantly reduced when SlEBF3-HA and SlBBX20-FLAG were co-expressed in tobacco (Fig. 5f). SlEBF3 is a substrate-recruiting subunit of Skp1-cullin1-F-box (SCF)-type E3 ubiquitin ligases that are responsible for binding and ubiquitinating degradation substrates. Therefore, we speculate that the interaction between SlEBF3 and SlBBX20 results in the degradation of SlBBX20 by ubiquitinated. To validate this hypothesis, we detected the degree of ubiquitination with an anti-ubi antibody and found that there was a higher degree of ubiquitination in the presence of SlEBF3 (Fig. 5f). To validate these results, Ub-HA was co-expressed with SlBBX20-FLAG in tobacco, and the result of the Co-IP experiments showed that Ub could be bound by SlBBX20-FLAG (Fig. 5g).

To investigate the ubiquitination mechanism of SlBBX20, we analyzed the protein sequence and identified two lysine residues (K143 and K152) that were predicted as potential ubiquitination sites (https://sumo.biocuckoo.cn/; Fig. S3b). To map the specific residues of SlBBX20 responsible for its SlEBF3-dependent ubiquitination and proteasomal turnover, we conducted a site-directed mutagenesis analysis of 2 lysine residues in the SlBBX20 protein (Fig. S3c). Co-expression of SlEBF3 with mutant SlBBX20 in tobacco leaves, the single mutations at K143 or K152 decreased the ubiquitination and increased the protein level of SlBBX20 (Fig. 5h). After mutating both K143 and K152, no ubiquitination of SlBBX20 was detected, and the protein level of SlBBX20 was twice as high as in the case of ubiquitination (Fig. 5h). To further demonstrate that the interaction between SlEBF3 and SlBBX20 promotes the degradation of SlBBX20, we performed protein degradation assays. Cycloheximide (CHX) was used in the co-expression system to assess the degradation rate of SlBBX20. SlBBX20 was rapidly degraded in the presence of SlEBF3-HA (Fig. 5i and j). In contrast, the degradation of SlBBX20 was greatly slowed when it was expressed alone (Fig. 5i and j). Taken together, these results demonstrate that SlEBF3 mediates the ubiquitin-dependent degradation of SlBBX20, thereby restoring the SlMED25-SlMYC2-mediated activation of SlJA2L. Furthermore, our mutagenesis analysis identified lysine residues K143 and K152 as critical sites for SlEBF3-mediated ubiquitination and subsequent degradation of SlBBX20.

### SlEBF3 inhibits *SlPSY1* activation by SlBBX20 and thus impacts phytoene synthesis

**Figure 6:**
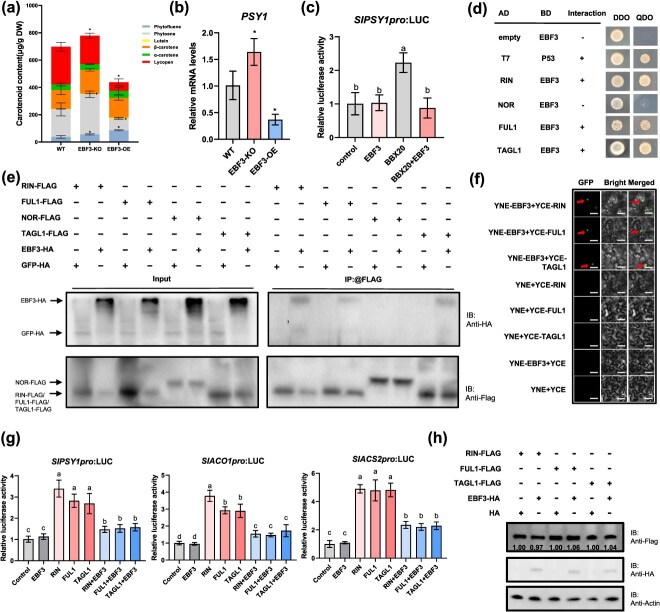
SlEBF3 involved in fruit ripening and carotenoid metabolism. (a) Carotenoid contents in fruits of the WT, *SlEBF3*-KO lines, and *SlEBF3*-OE at Br + 7 stage. Each value represents the mean of three biological replicates. Data are shown as means± SD. Asterisks indicate statistical significance using Student’s *t*-test, *P* < 0.05. (b) Relative expression levels of *SlPSY1* in WT, SlEBF3-KO, and SlEBF3-OE lines. Expression levels were normalized to untreated WT controls (set as 1) and *SlActin* was used as an internal control. Data are shown as means ± SD (*n* = 3). Asterisks indicate statistical significance using Student’s *t*-test, *P* < 0.05. (c) SlEBF3 represses the activation of *SlPSY1* by SlBBX20. The LUC/REN ratio represents the relative activity of the *SlPSY1* promoter. Data are shown as means ± SD (*n* = 3). Values marked with different letters indicate statistically significant differences as analyzed by a one-way ANOVA test from three independent biological replicate experiments, *P* < 0.05. (d) Screening for ripening regulators that interact with SlEBF3 by yeast two-hybrid. SlRIN, SlNOR, SlFUL1, and SlTAGL1 were used as bait, SlEBF3 was used as prey. Interactions between P53 and T7 and between SlEBF3-BD and empty AD were used as positive and negative controls, respectively. (e) *In vivo* Co-IP assays of SlEBF3 with SlRIN, SlNOR, SlFUL1, and SlTAGL1. SlRIN-FLAG, SlNOR-FLAG, SFUL1-FLAG, and SlTAGL1-FLAG were co-infiltrated with SlEBF3-HA in *N. benthamiana* leaves by Agrobacterium-mediated infiltration. Total proteins were extracted and immunoprecipitated with anti-FLAG antibody. GFP-FLAG was used as negative control. Immunoblots were probed with anti-FLAG antibody to detect SlRIN, SlNOR, SFUL1, SlTAGL1, and anti-HA antibody to detect SlEBF3. (f) BiFC assay of interaction between SlEBF3 and SlRIN, SlFUL1, and SlTAGL1. Bar = 50 μm. (g) Influence of SlEBF3-62sk, SlRIN-62sk, SlFUL1-62sk, and SlTAGL1-62sk on the expression of *SlPSY1*, *SlACO1*, and *SlACS2* in transient expression assays. The LUC/REN ratio represents the relative activity of the *SlPSY1*, *SlACO1*, and *SlACS2* promoter. Data are shown as means ± SD (*n* = 3). Values marked with different letters indicate statistically significant differences as analyzed by a one-way ANOVA test from three independent biological replicate experiments, *P* < 0.05. (h) Effect of SlEBF3 on the protein stability of SlRIN, SlFUL1, and SlTAGL1. The SlRIN-FLAG, SlFUL1-FLAG, and SlTAGL1-FLAG were expressed in the presence (+) or absence (−) of SlEBF3-HA in *N. benthamiana* leaves. Proteins were detected by immunoblotting and analyzed by immunoblotting with anti-FLAG, anti-HA, and anti-actin antibodies.

SlBBX20 was reported to regulate carotenoid metabolism by directly binding the promoter of *SlPSY1* and activating its expression [28]. However, whether the interaction between SlEBF3 and SlBBX20 influences SlBBX20-mediated transcriptional regulation of SlPSY1 is unclear. We determined the carotenoid content by liquid chromatography–mass spectrometry (LC–MS) and detected a significant increase in total carotenoid content in *SlEBF3*-KO and a notable decrease in *SlEBF3*-OE compared to the WT (Fig. 6a). Notably, the expression of PSY1, a key carotenoid biosynthetic gene, was significantly upregulated in the *SlEBF3*-KO lines but markedly downregulated in the *SlEBF3*-OE lines (Fig. 6b). A dual-luciferase reporter (DLR) assay was performed to investigate whether the physical interaction between SlEBF3 and SlBBX20 affects SlBBX20-mediated transcriptional regulation. The results of DLR indicated that SlBBX20 could activate *SlPSY1*, whereas SlEBF3 could inhibit the activation of *SlPSY1* by SlBBX20 (Fig. 6c). Taken together, these data suggest that SlEBF3 affects the accumulation of carotenoids by inhibiting the transcriptional activity of SlBBX20 on *SlPSY1* gene.

### SlEBF3 interacts with SlRIN, SlFUL1, and SlTAGL1 without undergoing ubiquitination and inhibits their upregulation of *SlPSY1*, *SlACO1*, and *SlACS2*

We have reported that SlEBF3 is involved in fruit ripening by regulating the accumulation of EILs [36]. However, it remains unclear whether SlEBF3 can regulate fruit ripening-related traits independently of its canonical role in EIL protein degradation. To explore whether there are other molecular mechanisms by which SlEBF3 regulates the fruit ripening process, we investigated the interactions between SlEBF3 and several ripening regulators. The yeast two-hybrid assay indicated that SlEBF3 interacts with SlRIN, SlFUL1, and SlTAGL1, but not with SlNOR (Fig. 6d). We performed a Co-IP assay after transiently co-infiltrating of SlEBF3-HA with SlRIN/SlNOR/SlFUL1/SlTAGL1-FLAG constructs in *N. benthamiana* leaves to validate these interactions *in vivo*. The findings indicated that SlEBF3-HA co-precipitated with SlRIN-FLAG, SlFUL1-FLAG, and SlTAGL1-FLAG, but not with SlNOR-FLAG, which aligns with the yeast two-hybrid results (Fig. 6e). Furthermore, direct physical interactions between SlEBF3 and SlRIN/SlFUL1/SlTAGL1 were confirmed through BiFC assay (Fig. 6f). These findings demonstrate that SlEBF3 interacts with SlRIN, SlFUL1, and SlTAGL1 both *in vitro* and *in vivo*.

*SlPSY1*, *SlACO1*, and *SlACS2*, which were significantly downregulated in SlEBF3-OE lines (Fig. S4), are direct targets of SlRIN, SlFUL1, and SlTAGL1. To investigate whether the interaction between SlEBF3 and SlRIN, SlFUL1 and SlTAGL1 affects the activation of their target genes, a DLR assay was conducted. It showed that SlBBX20 activate SlPSY1, while SlEBF3 inhibit the activation of *SlPSY1*, *SlACO1*, and *SlACS2* by SlRIN, SlFUL1, and SlTAGL1 (Fig. 6g). Taken together, these data suggest that SlEBF3 modulates carotenoid metabolism and fruit ripening by interacting with SlRIN, SlFUL1, and SlTAGL1 to suppress their transcriptional activation of the SlPSY1, SlACO1, and SlACS2 promoters.

Next, we wonder whether the interaction between SlEBF3 and SlRIN/SlFUL1/SlTAGL1 affects their protein abundance. After SlEBF3-HA was transiently co-expressing with SlRIN-FLAG, SlFUL1-FLAG, and SlTAGL1-FLAG in *N. benthamiana* leaves, we tested the accumulation of proteins, the results showed that SlEBF3 specifically reduced SlTAGL1 protein accumulation without affecting SlRIN and SlFUL1 protein levels (Fig. 6h). To verify whether ubiquitination occurs during the interaction of SlEBF3 with SlRIN/SlFUL1/SlTAGL1, we examined the ubiquitin binding of SlRIN-FLAG, SlFUL1-FLAG, and SlTAGL1-FLAG *in vivo* by Co-IP. Ub-HAs were co-expressed with SlRIN-FLAG, SlFUL1-FLAG, and SlTAGL1-FLAG in tobacco, and the results of the Co-IP experiments showed that SlRIN-FLAG, SlFUL1-FLAG, and SlTAGL1-FLAG could not be bound by Ub (Fig. S5a). Subsequently, we examined the protein stability of SlRIN/SlFUL1/SlTAGL1 under the influence of the proteasome inhibitor MG132. The data showed that MG132 treatment did not significantly affect the protein degradation rates of SlRIN, SlFUL1, and SlTAGL1 (Fig. S5b), suggesting that SlRIN, SlFUL,1, and SlTAGL1 may not be degraded through the 26S ubiquitin-proteasome system. The above results indicate that SlRIN, SlFUL1, and SlTAGL1 cannot be ubiquitinated *in vivo*. To further examine the functional interplay between EBF3 and transcription factors, we performed electrophoretic mobility shift assays (EMSA). And found that TAGL1 can bind to the *PSY1* promoter region alone; however, the binding of TAGL1 to the *PSY1* promoter was abolished upon the addition of EBF3 (Fig. S5c). These results indicate that the interaction between EBF3 and TAGL1 inhibits TAGL1’s ability to bind the promoter of its downstream target gene. Thus, we propose that EBF3 represses transcriptional activation of downstream target genes by inhibiting the transcription factor’s binding to their promoters.

### *SlEBF3*-OE fruits extend shelf life by increasing cuticle thickness and the content of cellulose in pericarps.

**Figure 7:**
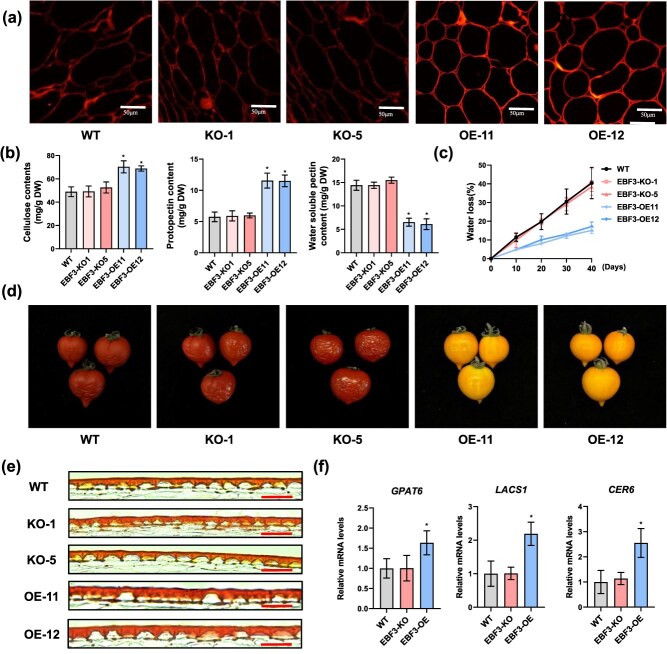
OE of *SlEBF3* prolongs fruit shelf life. (a) Pericarp sections stained with Congo red in WT, *SlEBF3*-KO, and *SlEBF3*-OE fruits. Bar = 50 μm. (b) Cellulose, protopectin, and water-soluble pectin contents in pericarps of WT, *SlEBF3*-KO1, *SlEBF3*-KO5, *SlEBF3*-OE11, and *SlEBF3*-OE12 fruits. Data are shown as means ± SD (*n* = 3). (c, d) Water loss in WT, SlEBF3-KO1, SlEBF3-KO5, SlEBF3-OE11, and SlEBF3-OE12 fruits. WT, SlEBF3-KO1, SlEBF3-KO5, SlEBF3-OE11, and SlEBF3-OE12 fruits harvested at BR + 10 stage and stored at room temperature. Photographs were taken at 40 d of storage. Bar = 10 mm. Data are shown as means ± SD (*n* = 15). (e) Cuticle deposition analysis in WT, *SlEBF3*-KO, and *SlEBF3*-OE11 fruits by Sudan IV staining. (f) Relative expression levels of cuticle synthesis–related genes *GPAT6*, *CER6*, and *LACS1* in WT, *SlEBF3*-KO, and *SlEBF3*-OE lines. The mRNA levels of each gene in WT were standardized to 1 and normalized with actin genes. Data are shown as means ± SD (*n* = 3). Asterisks indicate statistical significance using Student’s *t*-test, *P* < 0.05.

To assess the role of SlEBF3 in fruit shelf life, we initially measured the fruit firmness in WT, KO, and OE lines. No statistically significant variations in firmness were observed at the BR stage. However, the firmness of fruits from SlEBF3-OE lines was higher than that from WT and SlEBF3-KO lines at Br + 7 stage (Fig. S6). The cellulose content of the fruit is one of the most significant factors affecting fruit firmness [23]. To investigate whether *SlEBF3* affects cellulose content, we specifically stained cellulose in the cell wall with Congo red. The results showed that *SlEBF3*-OE fruits showed markedly higher cellulose fluorescence in cell walls than WT and *SlEBF3*-KO fruits (Fig. 7a). The contents of key cell wall components such as hemicellulose, total pectin, protopectin, and soluble pectin were determined (Fig. 7b, Fig. S7). Cellulose content was elevated in *SlEBF3*-OE fruits than in WT and *SlEBF3*-KO fruits, aligning with the observed cellulose fluorescence levels (Fig. 7b). The results showed that the contents of hemicellulose and total pectin in SlEBF3-KO and SlEBF3-OE did not change significantly compared with WT (Fig. S7). In SlEBF3-KO, the contents of protopectin and soluble pectin did not change significantly compared to WT, whereas in SlEBF3-OE, the contents of protopectin were higher and soluble pectin lower in the fruits of SlEBF3-OE compared to those of WT (Fig. 7b). Protopectin is water-insoluble and is known to increase fruit firmness, while soluble pectin is water-soluble, associated with reduced fruit firmness. These results indicate that SlEBF3 enhances fruit firmness by modulating cell wall composition.

Disease resistance and storage tolerance are both critical indicators of postharvest quality of tomato fruits, both of which significantly influence the shelf life of fruits. To investigate whether *SlEBF3* affects tomato fruit storage tolerance, we examined the role of *SlEBF3* in postharvest fruit storage. Fruits of WT, *SlEBF3*-KO, and *SlEBF3*-OE lines were collected at Br + 7 stage to assess water loss rate changes over 40 days postharvest at room temperature. The results showed that *SlEBF3*-OE fruits exhibited reduced pericarp shrinkage and withering, along with a lower water loss rate over 40 days compared to WT and *SlEBF3*-KO fruits (Fig. 7e and f). To further elucidate the potential mechanism by which SlEBF regulates fruit water loss, we examined cuticular deposition in fruits at Br + 7 stage by Sudan IV staining. *SlEBF3*-OE lines exhibited significantly increased cuticle thickness compared with WT, whereas *SlEBF3*-KO showed no difference (Fig. 7g). RT-qPCR was conducted to confirm the transcript levels of several key genes involved in cuticle synthesis such as ECERIFERUM6 (*CER6*), LONG CHAIN ACYL-COA SYNTHETASE 1 (*LACS1*), and GLYCEROL-3-PHOSPHATE ACYLTRANSFERASE 6 (*GPAT6*). The expression level of *SlGPAT6*, *SlLACS1*, and *SlCER6* were notably elevated in *SlEBF3*-OE fruits (Fig. 7f). These findings suggest that SlEBF3 promote the synthesis of fruit cuticles by increasing *SlGPAT6*, *SlLACS1*, and *SlCER6* expression, thereby reducing fruit water loss during storage.

## Discussion

**Figure 8:**
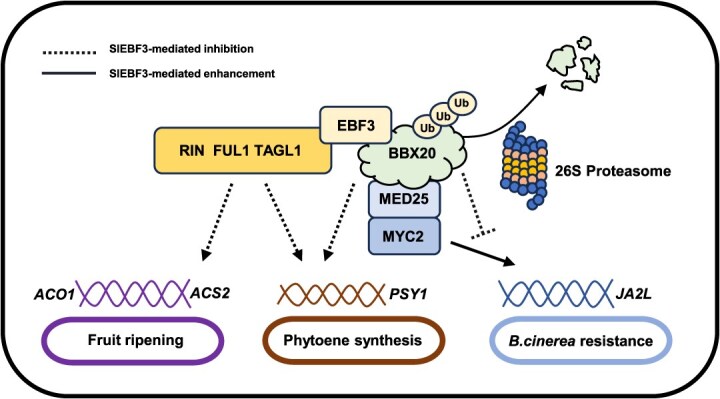
Working model of SlEBF3-mediated regulation of *B. cinerea* resistance, carotenoid biosynthesis, and fruit ripening in tomato.

EIN3-binding F-box proteins (EBFs) are crucial components in ET signaling. In Arabidopsis, EBF1 and EBF2 can target EIN3 and EIN3-like 1 (EIL1) for degradation. The functional roles of EBF1 and EBF2 are temporally distinct: EBF1 mediates early ET responses, whereas EBF2 contributes to subsequent signaling events [38–40]. In tomato, four *SlEBF* genes have been identified that have been demonstrated to play both overlapping and distinct roles in regulating ET signaling [20, 41]. While the function of SlEBF in the regulation of maturation has been largely elucidated, its importance in other biological processes remains uncertain. Here, we demonstrate that *SlEBF3* responds to JA signaling and enhances postharvest resistance to *B. cinerea* in tomato fruit by interacting with and suppressing SlBBX20, a known negative regulator of JA signaling and resistance to *B. cinerea* (Fig. 8). The interaction of SlEBF3 and SlBBX20 inhibited the accumulation of SlBBX20 and restored its inhibitory effect on the activation of *SlJA2L* by SlMED25-SlMYC2 to improve postharvest resistance in tomato fruit. Furthermore, our results demonstrate that SlEBF3 regulates carotenoid synthesis by inhibiting the activation of *SlPSY1* through interactions with SlBBX20, SlRIN, SlFUL1, and SlTAGL1. Additionally, SlEBF3 modulates fruit ripening by interacting with SlRIN, SlFUL1, and SlTAGL1 to suppress their activation of *SlACO1* and *SlACS2* (Fig. 8). In conclusion, our findings identify a new role for SlEBF3 in modulating JA signaling, enhancing *B. cinerea* resistance in tomato fruits and expanding the regulatory network for carotenoid metabolism and fruit maturation (Fig. 8).

SlEBF3 ubiquitinates and degrades SlBBX20, restoring its inhibitory effect on SlMED25-SlMYC2 activation of the JA-responsive gene SlJA2L, which then enhances the expression of *SlJA2L* and improves tomato resistance to *B. cinerea*. SlEBF3 also inhibits activation of *SlPSY1* by SlBBX20, which in turn inhibits phytoene synthesis. In addition, SlEBF3 can interact with SlRIN, SlFUL1, and SlTAGL1 without ubiquitination, but it can inhibit the activation of *SlPSY1*, *SlACO1*, and *SlACS2* by SlRIN, SlFUL1, and SlTAGL1 to regulate the synthesis and ripening process of phytoene in tomato fruit. SlEBF3 can also positively regulate cuticle thickness and cellulose content, thereby maintaining the postharvest quality of tomato fruits. Solid lines indicate SlEBF3-mediated enhancement, dashed lines indicate SlEBF3-mediated inhibition.

The relationship between EBFs exhibit considerable complexity in their regulatory networks [39, 42]. SlEBFs exhibit both overlapping and distinct functions in controlling tomato fruit ripening [41]. In this study, our data suggest that no statistically meaningful differences were detected in the transcriptional profiles of disease resistance, ripening, or cuticle-related genes between WT and KO plants (Figs 2g and 4b, c, andf). These findings suggest that SlEBFs exhibit complete functional redundancy in regulating disease resistance, ripening processes, and cuticle development. Notably, *SlPSY1* expression was significantly upregulated, accompanied by an increase in carotenoid content, in *SlEBF3*-KO fruit compared with WT fruit. In contrast, *SlPSY1* expression was downregulated and carotenoid content was decreased in *SlEBF3*-OE fruit (Fig. 5a and b). These results suggest that SlEBFs are not fully functionally redundant in the regulation of carotenoid metabolism. The redundant and specific functions of SlEBFs in different phenotypes demonstrate the flexibility and breadth of SlEBFs in the regulation of fruit ripening, metabolism, and postharvest quality, but the linkage and division of labour among SlEBF family members and the specific regulatory networks need to be further explored and validated. In conclusion, our results provide further evidence for the intricate regulatory relationships among SlEBF family members, revealing both significant functional redundancy and distinct functional specificity.

JA plays crucial roles in plant defense mechanisms [10, 43–45]. SlMYC2 is a core transcription factor of the JA signaling response that can interact with the multitalented Mediator subunit MED25 to form a transcriptional activation complex that activates the transcription of JA-responsive genes such as *SlJA2L* and *SlERF.C3*, which all positively regulate tomato *B. cinerea* resistance [10, 14, 15]. This study shows that SlEBF3 positively regulates the JA response to *B. cinerea* infection. The expression of *SlEBF3* was significantly upregulated after MeJA treatment (Fig. 3a), indicating that *SlEBF3* can be induced by JA. The *SlEBF3*-OE strain showed a significant induction in the expression of JA-responsive genes *SlJA2L* and *SlTD* (Fig. 3b and c). Transcriptomic analysis revealed an up-regulation of JA signaling and response genes in *SlEBF3*-OE following *B. cinerea* inoculation (Fig. 2c). Collectively, these findings indicate that *SlEBF3* enhances the JA signaling pathway to regulate gray mold resistance.

F-box proteins are crucial elements of SCF (Skp1-Cullin1-F-box) E3 ubiquitin ligase complexes, and act as specificity determinants for substrate recognition. These proteins catalyze the polyubiquitination of specific substrates, leading to their eventual destruction via the 26S proteasome pathway. Through this regulatory mechanism, F-box proteins significantly influence diverse physiological processes, particularly in plant hormone signaling pathways and immune response regulation [46]. During ET signaling, EIN2 and EIN3 are regulated by their respective F-box proteins, ETP1/2 and EBF1/2, whereas EIL1-4 can be degraded by EBF3 in tomato [36, 38–40]. In auxin signal transduction, Aux/IAA proteins are targeted by TIR1/AFB F-box proteins, leading to their ubiquitination and subsequent degradation via the SCF-26S proteasome system [47–50]. In the JA signaling pathway, COI1 can mediate ubiquitination degradation of the JAZ repressor, thereby derepressing MYC2 [12–14]. SlBBX20 can be modified by ubiquitination and can negatively regulate the JA signaling pathway and *B. cinerea* resistance. In addition, SlBBX20 can interact with eight SlJAZ proteins and SlJAZ proteins can also interact with SlMTBs (MYC2 TARGETED BHLH) [16, 29]. In this study, SlEBF3 can affect JA signaling and thus regulate *B. cinerea* resistance by binding and degrading SlBBX20. Whether SlEBF3 can affect other components of the transcriptional repressor complex consisting of SlBBX20, SlMTB, and SlJAZ proteins in the same way needs to be further investigated and demonstrated. In addition, SlEBF3 was able to interact with SlRIN, SlFUL1, and SlTAGL1 without undergoing ubiquitination (Fig. 6), which is a novel pathway for SlEBF3 to regulate maturation without degrading SlEILs proteins. Further research and verification are needed to elucidate the molecular mechanisms by which SlEBF3 affects the activation of target genes by SlRIN, SlFUL1, and SlTAGL1.

Both postharvest resistance and storage tolerance of tomato fruit are important factors in maintaining postharvest fruit quality. Excessive postharvest softening of fruit results in reduced resistance to pathogens and increased water loss, which in turn leads to fruit deterioration [51]. We found that SlEBF3-OE fruits could reduce water loss and extend shelf life by altering cell wall composition (Fig. 7), and that postharvest changes in fruit texture and disease resistance were closely related, with specific linkages that need to be further explored in future studies. Overall, our study revealed the molecular associations and potential mechanisms by which SlEBF3 affects JA signaling during postharvest resistance to *B. cinerea* in tomato fruits, and also enriched the regulatory network of SlEBF3 affecting the ripening process and carotenoid metabolism in tomato fruits. In addition, *SlEBF3* could be a potential candidate gene for the genetic improvement of fruit postharvest disease resistance and storage tolerance to extend fruit shelf life.

## Materials and methods

### Plant materials

Tomato plants ‘MicroTom’ (MT) was used as WT. Three *SlEBF3*-OE lines were obtained from *Deng* [36]. Independent guide RNA of *SlEBF3* were designed using the application tool CRISPR-P and inserted into the plasmid of pFASTCas9/ccdB to construct the CRISPR/Cas9 genome editing plasmid vector of *SlEBF3*. The recombinant plasmid pFASTCas9/ccdB-SlEBF3 was verified through Sanger sequencing before being introduced into ‘MicroTom’ tomato plants using Agrobacterium-mediated transformation [21].

### *B. cinerea* infection assay

Ten tomato fruits of each group at MG, Br, and Br + 7 stages were punctured with a sterile needle, and then the mycelial plugs were placed over the wound with mycelial plugs from the edge of fungal cultures. For leaf incubation, mycelial plugs were applied to the front side of 35- to 45-day-old leaves that has been pierced with an anatomical needle. Inoculated fruits and leaves were kept at 20°C in darkness. Diameter of each lesion on fruits or leaves were measured 48 or 96 h postincubation (hpi).

### Measuring fruit firmness

For WT and each transgenic line, a minimum of 15 fruits were randomly selected at both the Br stage and Br + 7 stage. Fruit firmness measurements were conducted with the TA.XTC-18 (BOSIN, Shanghai).

### Histological analysis of fruit pericarp

For cellular morphology analysis, equatorial sections (3 mm in thickness) of breaker-stage (Br) fruits were prepared. After brief staining with 0.5% toluidine blue (30 s), samples were photographed using a stereomicroscope (M205FA, Leica, Germany). Fruits at Br and Br + 7 stages were collected for cell wall characterization. Sequential staining with safranin O and fast green for 4–6 s each preceded neutral balsam mounting and brightfield microscopy (Olympus VS200, Japan). Cellulose detection was achieved through Congo Red (Servicebio, China) staining of paraffin sections, with fluorescence visualization using a Leica DM4 B microscope (Germany). Quantitative analysis of cellular parameters (including cell number, size, and pericarp thickness) was conducted utilizing ImageJ.

### Measuring fruit cellulose, hemicellulose, total pectin, protopectin, and soluble pectin contents

At least 10 fruits per line were harvested at the Br + 7 stage. Samples were snap frozen in liquid nitrogen and ground into powder. Three portions of cell wall material were weighed, and cellulose, hemicellulose, total pectin, protopectin, and soluble pectin contents were measured using a Cell Wall Assay kit (Solarbio, Beijing) according to Pei *et al*.

### Quantification of postharvest water loss

A shelf-life analysis was conducted on tomato fruits from three genetic backgrounds: WT, SlEBF3-KO, and SlEBF3-OE. Fifteen fruits of each line were stored in 22°C. Fruits weight was recorded initially and then every 10 days over a 40-day period. Water loss was quantified as the percentage decrease in fruit mass relative to the initial weight [52].

### Measuring fruit color

Fruits from WT, *SlEBF3*-KO, and *SlEBF3*-OE fruits were collected at Br + 7 stage. Fruit color was quantitatively measured with a Konica Minolta chromameter (CR-400; Konica Minolta, Tokyo, Japan) following established protocols [53].

### Measuring carotenoid contents

Carotenoid content in WT, *SlEBF3*-KO, and *SlEBF3*-OE fruits at the Br + 7 stage were measured following the method outlined by You [54]. Freeze-dried fruit powder (100 mg) from different samples was extracted with a mixture of n-hexane, acetone, and ethanol (2:1:1, v/v/v). Each sample was vortexed briefly, sonicated at 25°C for 20 min, centrifuged (13 000 g), and the supernatant collected; this process was repeated one time. The supernatants were concentrated in a Concentrator Plus (Eppendorf) and reconstituted in methanol and 1% (v/v) methylene chloride. Carotenoid content was determined by LC–MS. Carotenoid composition was determined by HPLC analysis using authentic standards.

### RNA extraction and RT-qPCR

Following the manufacturer’s instructions, total RNA was isolated from fruit pericarp and leaves with a TRIzol Reagent Kit (Takara, Otsu, Japan). A HiScript II 1st Strand cDNA Syn-thesis Kit (Vazyme, Nanjing, China) was used to reverse transcribe RNA into cDNA. The primer sequences used in this assay are listed in Dataset S1.

### Yeast two-hybrid assay

The coding sequences for *SlEBF3* was cloned into pGBKT7 and the coding sequences for *SlBBX20*, *SlRIN*, *SlNOR*, *SlFUL1*, *SlTAGL1*, *SlMYC2*, and *SlMED25* were cloned into pGADT7. Then transformed the AD and BD plasmids into AH109 yeast competent cells and screened on SD medium without Leu and Trp (SD-LW) for 3d. Interaction was detected in SD-4 (SD-LWAH) medium.

### Co-IP assay

The complete coding sequences of *SlBBX20*, *SlRIN*, *SlNOR*, *SlFUL1*, and *SlTAGL1* were cloned into the pBTEX-FLAG vector, while *SlEBF3* was cloned into the pBTEX-HA vector, resulting in the creation of transient expression vectors: SlBBX20-FLAG, SlRIN-FLAG, SlNOR-FLAG, SlFUL1-FLAG, SlTAGL1-FLAG, and SlEBF3-HA, respectively. The constructs were transiently co-expressed in *N. benthamiana* leaves, which were harvested 48–72 h post-infiltration. Immunoprecipitation was performed using FLAG M2 magnetic beads (Sigma, M8823) at 4°C for 16 h. Proteins were examined followed by 4-h incubation with antibodies (anti-HA #2367 or anti-FLAG #14793; Cell Signaling Technology).

### Electrophoretic mobility shift assay

Electrophoretic mobility shift assays (EMSAs) were performed as previously described [21–23]. The full-length coding sequence of SlTAGL1 was cloned into pet28a and the full-length coding sequence of SlEBF3 was cloned into pGEX-4 T-1. SlTAGL1-His and SlEBF3-GST recombinant proteins were produced in *E. coli* BL21 (DE3) cells. Proteins were purified using His-tag Protein Purification Kit (Beyotime, #P2247) and GST-tag Protein Purification Kit (Beyotime, #P2262). Probes containing probes from the SlPSY1 promoter were labeled with biotin using the 3′- Biotin Labeling Kit (Thermo Fisher; 89 818). EMSA experiments were subsequently performed using the EMSA/Gel-Shift kit (Beyotime) and the LightShift Chemiluminescent EMSA kit (Thermo).

### *In vivo* ubiquitination assay

The assay of ubiquitination *in vivo* was performed as described by Wang [55]. The two predicted lysine (K) sites were mutated individually and together to arginine (R). SlBBX20^K143R^-Flag, SlBBX20^K152R^-Flag, and SlBBX20^K143R/K152R^-Flag were cloned into pBTEX-FLAG. Tobacco leaves were used for co-expression assays. Anti-FLAG (CST, #14793), anti-HA (CST, #2367), and anti-ubiquitin (Abmart, PL026378S) were used to test the protein signal.

### Protein accumulation assays

The assay of protein stability and degradation were performed according to the experimental protocol of Wang *et al*. with some improvements [55]. For protein stability analysis, leaf tissues were harvested 36 h post-infiltration, treated with 50 μM MG132 to protein extraction. To assess degradation kinetics, leaves were exposed to 250 μM CHX at 36 hpi and sampled after 2-h incubation. Immunoblotting was performed using anti-FLAG (#14793; CST) and anti-HA (#2367; CST) antibodies. Protein band densitometry was performed using ImageJ analysis software, with final values expressed as mean ± SD derived from triplicate experimental replicates.

### Luciferase reporter assay

The full-length CDS of *SlEBF3*, *SlMYC2*, *SlMED25*, *SlBBX20*, *SlRIN*, *SlFUL1*, and *SlTAGL1* were cloned into the pGreenII 62sk vector as effectors. The promoter sequences of *SlJA2L*, *SlPSY1*, *SlACO1*, and *SlACS2* were cloned into pGreen-Mini35S-LUC vectors as reporters. The experimental procedures were conducted as previously described [56]. Vectors were introduced into *N. benthamiana* using GV3101. Transient expression was then conducted in tobacco leaves. The Dual-Luciferase Reporter Assay System (Promega, E1910) was employed to assess LUC and REN activities.

### Bimolecular fluorescence complementation

The BiFC method for studying protein–protein interactions utilized the pSPYCE and pSPYNE vectors as described in prior research [57]. The coding sequences of the *SlEBF3* and *SlBBX20*, *SlRIN*, *SlFUL1*, and *SlTAGL1* were inserted into the pSPYNE and pSPYCE plasmid. The resulting cultures were used for co-expression in *N. benthamiana* leaves. YFP fluorescence signal was observed and imaged using the fluorescence microscope (DM4 B, Leica, Germany).

### MeJA treatment

Four-weeks-old tomato leaves from WT and transgenic lines were treated with 100 μM MeJA. The leaves were collected after 1 h. The transcription levels of *SlEBF3*, *SlMYC2* and *SlJA2L* was quantified using RT-qPCR. (Table S1).

### Wheat germ agglutinin and propidium iodide staining

As described by Fernandez-Alvarez, we visualized fungal hyphae in planta by wheat germ agglutinin and propidium iodide (WGA-Pl) staining to evaluate the severity of fruits infection by *B. cinerea* [58]*.* Following 48-h *B. cinerea* infection, fruit pericarp tissues were preserved in Carnot’s fixative. Tissue sections were subsequently stained with a WGA-PI dual staining solution, then imaged using an Olympus VS200 digital slide scanning system (Olympus Corporation, Japan).

### Antioxidant enzyme activity

The enzymatic activities of antioxidant enzymes were assessed with detection kits (Solarbio, Beijing). Absorbance was read at 560 nm (SOD) and 470 nm (POD) wavelengths using a Thermo Multiskan GO.

### RNA-Seq analysis

RNA was extracted from WT and *SlEBF3*-OE fruits at the BR stage after *B. cinerea* inoculation, using three biological replicates for each genotype. Purified RNA samples were subsequently processed for cDNA library preparation and sequenced using MGI-DNB Seq platform (Shenzhen, China). DEGs were identified using a threshold of *P* < 0.05 and |FC| ≥ 1.5 [59].

### Statistical analysis

All data were analyzed using GraphPad Prism version 9. Results are expressed as mean values ± standard deviation (SD) from a minimum of three separate experiments. Intergroup comparisons were analyzed by Student’s *t*-test, with statistical significance set at *P* < 0.05 (denoted by asterisks).

## Supplementary Material

Web_Material_uhaf219

## Data Availability

The authors confirm that all the experimental data are available and accessible via the main text and/or the supplemental data.
