# Supplementary material for: EIN3-binding F-box protein SlEBF3 modulates resistance against Botrytis cinerea and carotenoid biosynthesis by degradation of BBX20 in tomato
Source: Hortic Res. 2025 Aug 22;12(11):uhaf219. doi: 10.1093/hr/uhaf219 (PMC12581942; doi:10.1093/hr/uhaf219)
Supplement: Web_Material_uhaf219 [file web_material_uhaf219.zip › Supplementary figures.pdf]

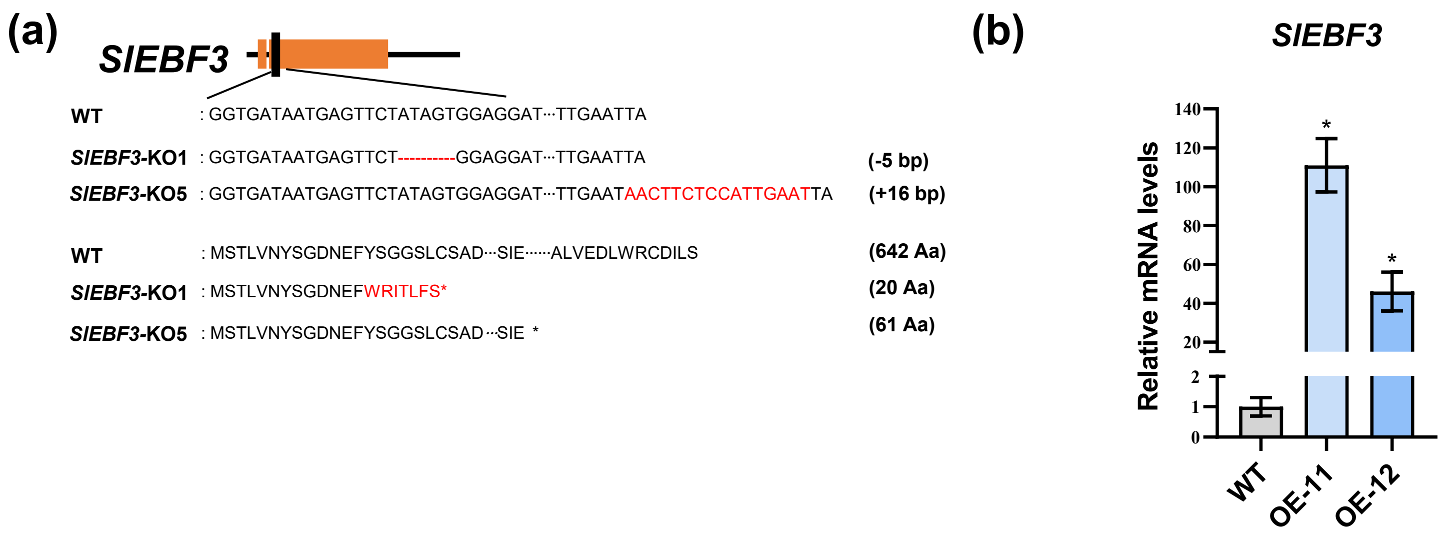

Supplemental Figure S1 Identification of *SlEBF3* mutants and overexpression lines

(a) Identification of *SlEBF3* mutants. *SlEBF3*-KO1and *SlEBF3*-KO5 are distinct mutants created through CRISPR/Cas9 genome editing. The red horizontal line represents the deletion mutation. The red letters represents the insertion mutation. **(b)** RT-qPCR analysis of *SlEBF3* expression level in *SlEBF3*-OE leaves. *SlEBF3*-OE11, *SlEBF3*-OE12 represent two independent lines. Relative mRNA levels of SlEBF3 in wild-type leaves were normalized to 1, and SlActin was used as an internal control. The data represent means ± SD of four biological replicates. *, $P < 0.05$ (Student's t-test).

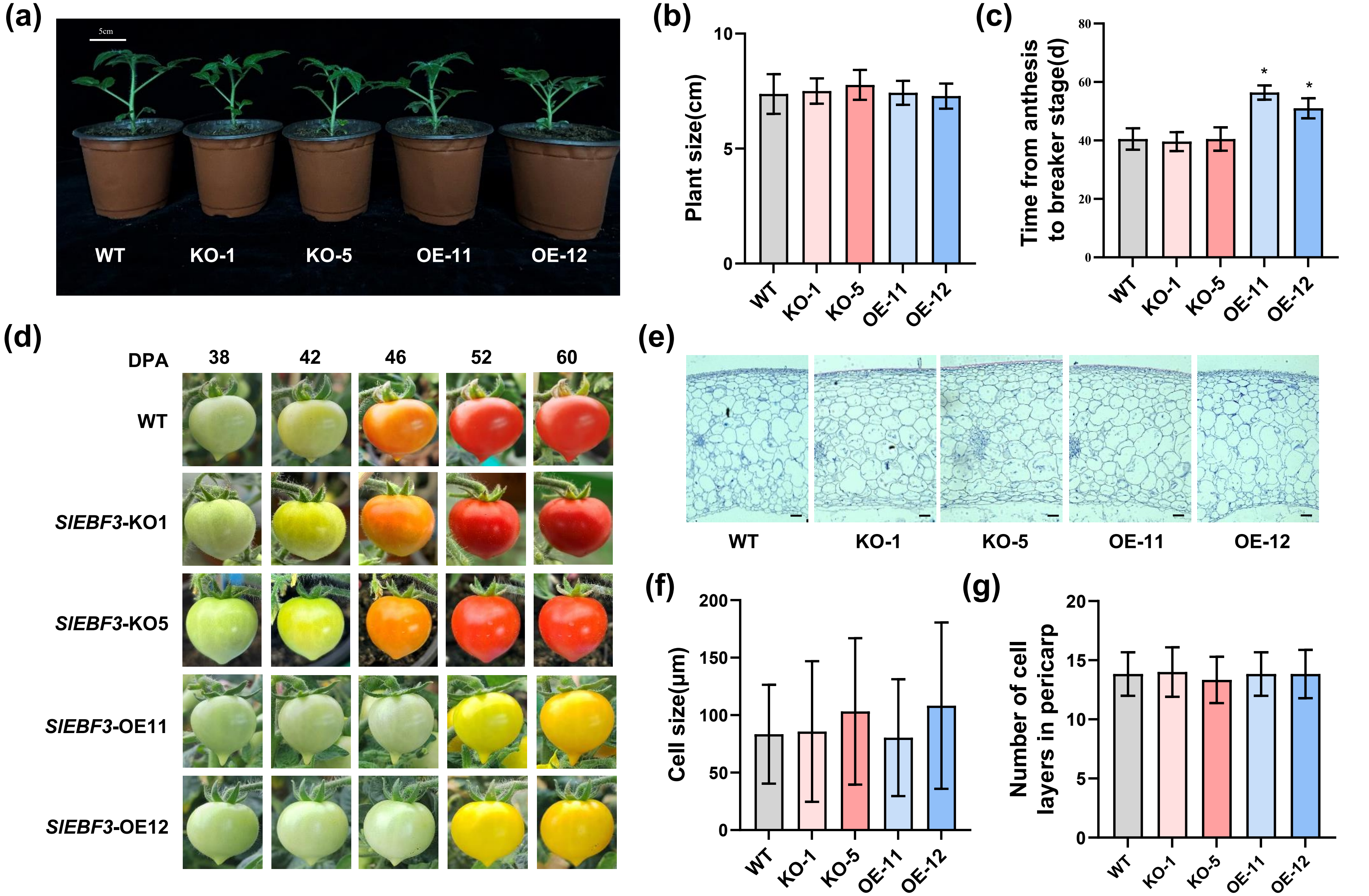

Supplemental Figure S2. Plant development and fruit ripening-related phenotypes of WT, *SlEBF3*-KO and *SlEBF3*-OE lines.

(a) Plants were photographed after 4 weeks of growth. Bar = 5 cm. **(b)** Plant sizes of WT, *SlEBF3*-KO1, *SlEBF3*-KO5, *SlEBF3*-OE11 and *SlEBF3*-OE12 lines. Data are shown as means ± SD (n = 8). **(c)** Time period from anthesis to Br in WT, *SlEBF3*-KO1, *SlEBF3*-KO5, *SlEBF3*-OE11 and *SlEBF3*-OE12 lines. Data are shown as means ± standard deviation (SD) (n = 10). Asterisks indicate statistical significance using Student's t-test, $P < 0.05$. **(d)** Fruit ripening process of WT, *SlEBF3*-KO1, *SlEBF3*-KO5, *SlEBF3*-OE11 and *SlEBF3*-OE12 lines. DPA, days post anthesis. **(e)** Sections of fruits stained with toluidine blue in WT, *SlEBF3*-KO1, *SlEBF3*-KO5, *SlEBF3*-OE11 and *SlEBF3*-OE12 lines at BR stage. Bar = 100μm. **(f)** Cell size in pericarp of WT, *SlEBF3*-KO1, *SlEBF3*-KO5, *SlEBF3*-OE11 and *SlEBF3*-OE12 lines. Data are shown as means ± SD (n = 3). **(g)** Number of cell layers in pericarp of WT, *SlEBF3*-KO1, *SlEBF3*-KO5, *SlEBF3*-OE11 and *SlEBF3*-OE12 lines. Data are shown as means ± SD (n = 3).

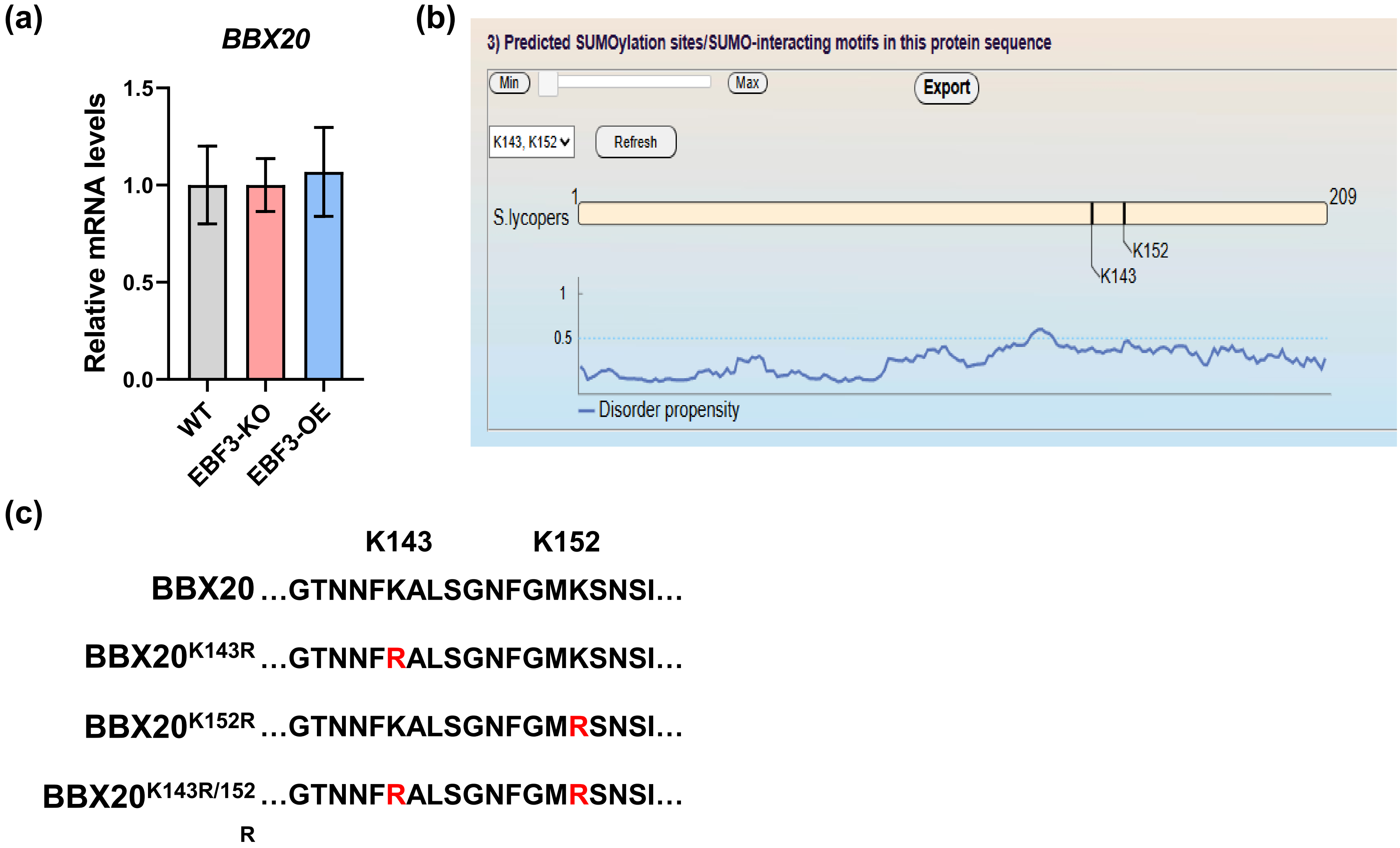

Supplemental Figure 3. RT-qPCR analysis, ubiquitination site identification, and mutant sequence verification

(a) Relative expression levels of *SlBBX20* in WT,*SlEBF3*-KO and *SlEBF3*-OE lines. Expression levels were normalized to untreated WT controls (set as 1) and *SlActin* was used as an internal control. Data are shown as means ± SD (n = 3), Asterisks indicate statistical significance using Student's t-test, P < 0.05. **(b)** Online analysis of potential ubiquitination sites of SlBBX20 protein (https://sumo.biocuckoo.cn/). **(c)** Protein sequences of wild type and mutants.

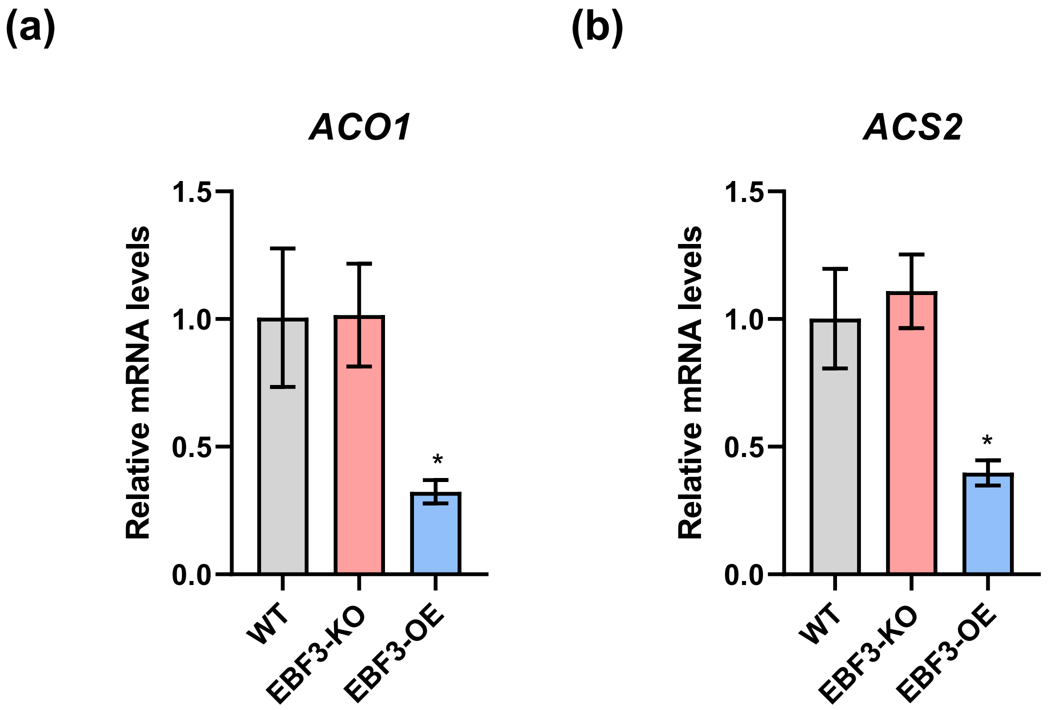

Supplemental Figure 4. Relative expression levels of *SlACO1* and *SlACS2* in WT,*SlEBF3*-KO and *SlEBF3*-OE lines.

Expression levels were normalized to untreated WT controls (set as 1) and *SlActin* was used as an internal control. Data are shown as means ± SD (n = 3), Asterisks indicate statistical significance using Student's t-test, P < 0.05.

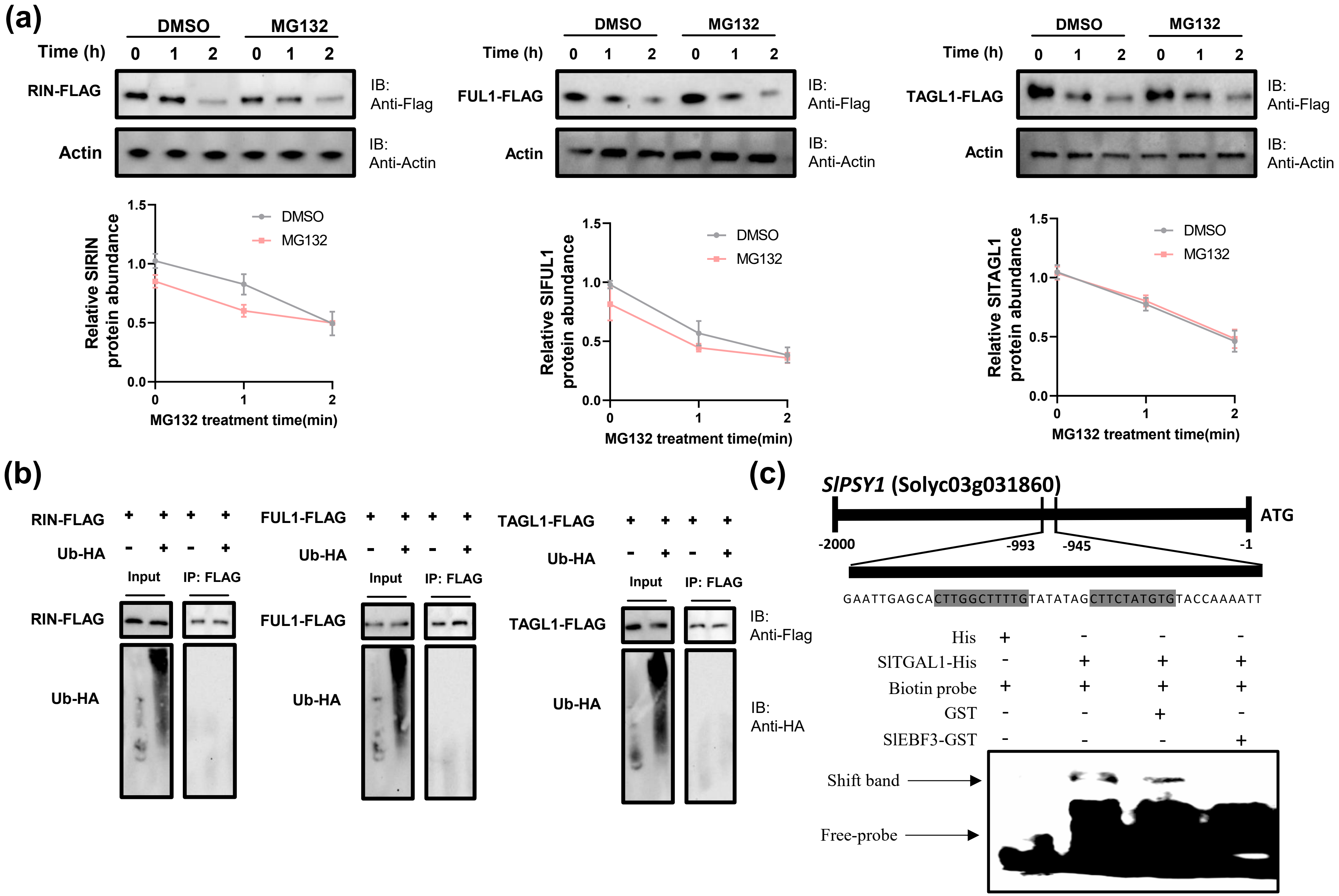

Supplemental Figure 5. SlRIN, SlFUL1 and SlTAGL1 are not degraded by the 26S proteasome-dependent pathway.

(a) Stability analysis of SlRIN,SlFUL1and SlTAGL1. SlRIN-FLAG, SlFUL1-FLAG and SlTAGL1-FLAG was expressed in N. benthamiana leaves followed by treatment with or without the proteasome inhibitor MG132. DMSO was used as a control. Total protein extracted from the transformed leaves was subjected to immunoblotting analysis using anti-HA or anti-Flag antibodies. SlRIN-FLAG, SlFUL1-FLAG and SlTAGL1-FLAG proteins were detected by immunoblotting. Proteins were analyzed by immunoblotting with anti-FLAG and anti-actin antibodies. quantification of the immunoblot bands were performed by Image J software. Values are means±standard deviation (SD) of 3 independent experiments. **(b)** In vivo detection of ubiquitination of SlRIN-FLAG, SlFUL1-FLAG and SlTAGL1-FLAG by Co-IP. **(c)** EMSA showed that SlEBF3 inhibits the direct binding of SlTAGL1 to the SlPSY1 promoter sequence in vitro.

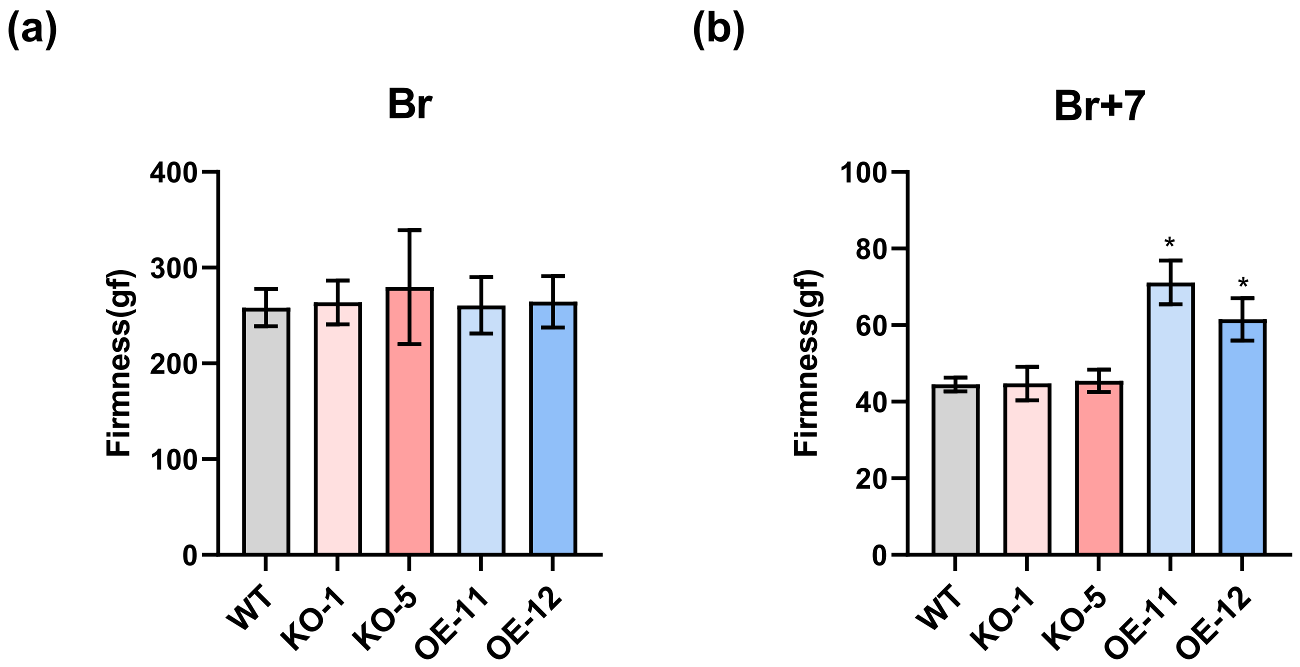

Supplemental Figure 6. Firmness of WT, *SlEBF3*-KO1, *SlEBF3*-KO5, *SlEBF3*-OE11 and *SlEBF3*-OE12 fruits at Br and Br + 7 stages.

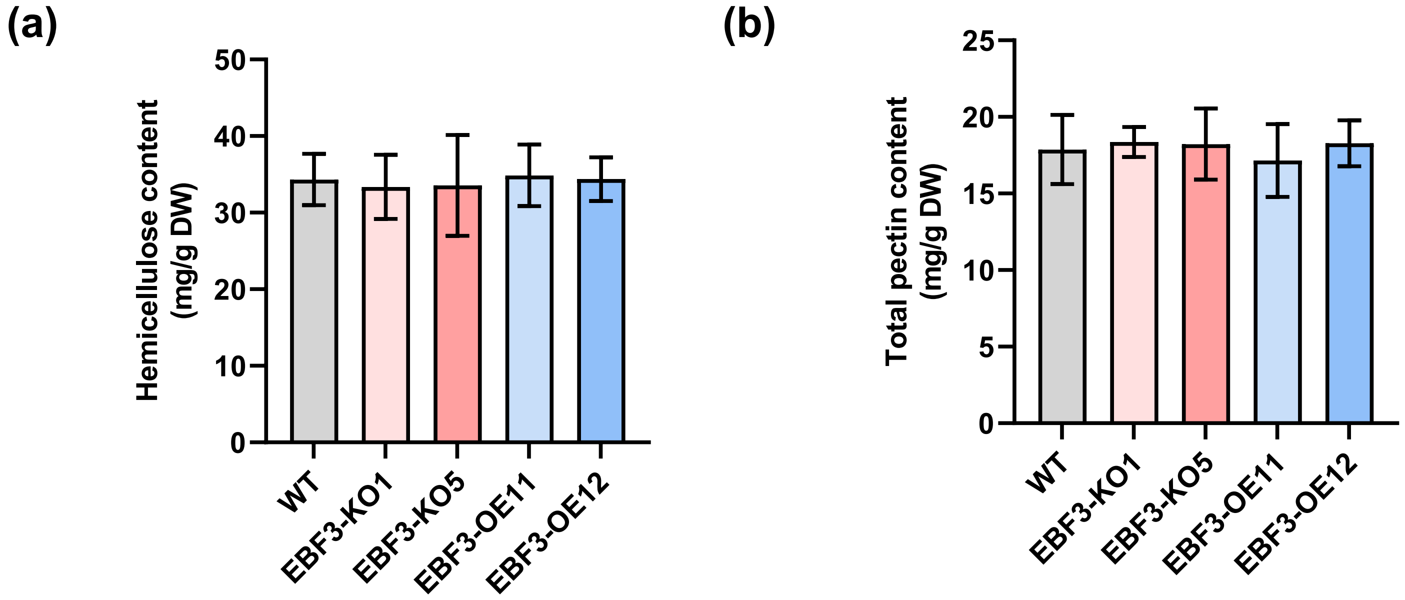

Supplemental Figure 7. Hemicellulose and total pectin contents in pericarps of WT, *SlEBF3*-KO1 , *SlEBF3*-KO5, *SlEBF3*-OE11 and *SlEBF3-OE12* fruits.
